# Fostering collaboration through learning communities: a case report on engaging with *All of Us* data among library professionals, faculty, and students

**DOI:** 10.5195/jmla.2026.2335

**Published:** 2026-07-01

**Authors:** Zachary McNiece, Dawn Hackman, Nick Szydlowski, Yuqi He

**Affiliations:** 1 zachary.mcniece@sjsu.edu, Assistant Professor, Department of Counselor Education, San José State University, San Jose, CA; 2 dawn.hackman@sjsu.edu, Health Sciences and Scholarly Communications Librarian, Dr. Martin Luther King Jr. Library, San José State University, San Jose, CA; 3 nick.szydlowski@sjsu.edu, Digital Scholarship Librarian, Dr. Martin Luther King Jr. Library, San José State University, San Jose, CA; 4 yuqi.he@sjsu.edu, Engineering and Data Services Librarian, Dr. Martin Luther King Jr. Library, San José State University, San Jose, CA

**Keywords:** All of Us, Interdisciplinary Collaboration, Data Services, Learning Communities, Group Learning, Teaching-focused Institutions, Less-resourced Institutions

## Abstract

**Background:**

Research data services (RDS) have expanded in academic libraries but can be challenging to develop, particularly in teaching-intensive and less-resourced institutions. Learning communities offer a promising model for building skills, fostering collaboration, and aligning services with local needs.

**Case Presentation:**

This case report describes the development and implementation of three learning communities—a library group, a faculty group, and a student group—at a teaching-focused institution. These communities brought together library professionals, faculty, and students from diverse disciplines—including health sciences, education, data science, and engineering—to collaboratively explore the All of Us dataset. By working with the same dataset, participants were able to move quickly from abstract concepts to hands-on practice, while developing a shared understanding of tools, workflows, and challenges. The learning communities also served as platforms for building institutional capacity in data-intensive research.

**Conclusions:**

The learning communities model proved to be an effective strategy for fostering cross-disciplinary collaboration, promoting data literacy, and building institutional readiness to support research using the All of Us dataset. By centering on local expertise, learning communities provide a sustainable, resource-conscious framework for developing RDS. This approach also demonstrates how academic libraries can act as conveners and catalysts for equitable data engagement. Lessons learned from this case may inform similar efforts at other institutions seeking to build collaborative, inclusive models for engaging with various data resources.

## BACKGROUND

Libraries’ research data services have expanded over the past decade, responding to changes in research methods and expanding data sharing requirements [1,2]. However, notable disparities in data service offerings exist among different types of institutions. A 2024 report by Ithaka S+R [3] finds that while general research data services are commonly offered across all institution types, more specialized services, such as statistical support, Geographic Information System (GIS), and visualization are more prevalent at research-intensive universities than liberal arts colleges or teaching-focused institutions. Common barriers to developing data services include limited staff, lack of internal expertise, limited financial resources, and minimal institutional support [2,4–5]. While Cox et. al [5] propose a maturity model outlining progressive stages in the development of data services and associated skills, not all institutions are positioned to follow the same path to maturity. Libraries servicing less-resourced institutions and user communities must find creative ways to meet growing researcher demand without overextending existing staff and resources.

Learning communities–time-bound commitments of active participation in collaboration and shared learning–are a common approach for capacity building and professional development in higher education [6]. To be sustainable, learning communities should ideally be built around a shared goal, comprise 8-12 participants, and require a time commitment of no more than a couple of hours per week [7]. Learning communities are grounded in social learning theory [8] and often andragogy, which positions community members as self-directed, intrinsically-motivated learners with previous experiences that support their learning and application of knowledge [9]. Participants’ intrinsic motivation to learn is what makes learning communities effective; a group comes together for a defined goal and period of time, with defined responsibilities to each other. These tasks become the source of the learning and the application of new information. Learning communities have been used for a variety of purposes, like improving student outcomes via teaching methods and developing best practices for using online learning management systems; learning communities have also been used within library-specific contexts, such as developing librarian competencies and supporting data literacy through librarian-facilitated faculty learning communities [10–14].

## CASE PRESENTATION

Established as a teaching college in 1857, San José State University (SJSU) is an Asian American and Native American Pacific Islander-Serving Institution and a Hispanic Serving Institution currently enrolling more than 36,000 students. While the primary focus of the institution continues to be on teaching and learning, SJSU has increased its focus on research capacity in recent years, culminating in a change in Carnegie Classification to R2 in 2025 [15]. This shift has increased the need for research data services and other library services that support research, yet resources to address this need are limited. This case presentation focuses on strategies for building capacity for research data services at a less-resourced institution that traditionally has not prioritized library support for research.

SJSU centers communities of practice in various institutional processes, including orientation for new faculty and learning communities in connection with programmatic grants to deepen interdepartmental collaboration and further innovative work. Faculty and students are accustomed to contributing effectively to communities of practice. The National Institutes of Health (NIH) *All of Us* Research Program is a particularly promising focal point for this approach, as it is a single data source that is relevant to researchers in a variety of disciplines.

Founded in 2016, the NIH *All of Us* Research Program's purpose is to improve healthcare through precision medicine research by connecting researchers to a large cohort of research participants from across the United States [16]. The *All of Us* Research Program dataset, accessible exclusively through the Researcher Workbench cloud platform, includes many types of data which can be used in health-related research, including surveys, electronic health records, biosamples, physical measurements, and wearables [17]. The *All of Us* Research Program has conducted extensive outreach to the research community to increase participation by groups of researchers from demographics underrepresented in the biomedical workforce (UBW) [18]. One part of these efforts was offering competitive awards to support capacity-building at institutions with a track record of supporting and educating UBW researchers.

At SJSU, external funding was used to build institutional capacity through the creation of three learning communities. A library-based learning community was supported by a $40,000 grant and train-the-trainer style education program from the NLM. This program allowed three librarians to build their expertise in accessing and using the Workbench. Parallel learning communities for faculty and students were established through a $75,000 grant from the NIH. An overarching goal of these learning communities was to promote collaboration between researchers in the health sciences and those in data science or other fields where the technical skills needed to work in the Researcher Workbench are widespread. These learning communities provided a site for training and skill-sharing in support of faculty and student research, helping address some of the challenges that individual researchers may face in attempting to adopt *All of Us* in their work.

The learning communities had two discrete objectives:

- to increase data-driven human health research conducted at SJSU by building the knowledge and skills needed to use the NIH *All of Us* Researcher Workbench; and
- to build institutional capacity of the SJSU Library for supporting data-intensive research by developing data knowledge and skills of librarians and library staff.

### Challenges Using *All of Us* at SJSU

The *All of Us* Researcher Workbench consists of a secure, browser-based environment where investigators can query *All of Us* data to build research cohorts and analyze data in a controlled environment using a set of available tools. For many researchers, working with data in the *All of Us* environment may require specific technical and data analysis skills that have not been required in their previous research work. *All of Us* data is complex, and researchers accustomed to working with smaller datasets may find the process of defining research cohorts and selecting data elements challenging and unfamiliar. Using the Researcher Workbench also requires significant technical skill; until the integration of SAS Studio in 2024, all research conducted using *All of Us* data required proficiency in Jupyter Notebook along with Python or R programming languages. SJSU health researchers more often use point-and-click software, such as SPSS or SAS, and may be unfamiliar with programming interfaces and coding. Further, due to the enormous size of the dataset across the number of participants and variables, it is not possible to “view the data” in the traditional sense. Researchers must utilize several internal and public-facing *All of Us* resources to develop a mental map of the dataset so they can wrangle the data and select the variables and participants of interest.

### Description of Approach

SJSU’s *All of Us* Institutional Champion Team is composed of three librarians, Engineering and Data Services Librarian (YH), Health Sciences Librarian (DH), and Digital Scholarship Librarian (NS), and one faculty member (ZM), an assistant professor in the Department of Counselor Education who already had experience with *All of Us* data. The group first formed through a pre-established connection from a previous learning community focused on digital counter-storytelling. Through initial conversations, email mailing lists, and direct communications, the group learned of funding opportunities that led to the present collaboration.

The team first applied for NIH’s *All of Us* Institutional Champion Award, and later the National Library of Medicine’s (NLM) *All of Us* Data Training and Engagement for Academic Libraries Program. These awards funded the creation of three distinct learning communities to engage with the *All of Us* data, which ran from February 2024 through February 2025. The projects' joint leadership ensured alignment across the learning communities and allowed for the integration of library resources and research support into both faculty and student learning communities.

The primary activities of the library learning community were conducted during the summer of 2024, providing greater flexibility and availability for professional development. The learning community was initiated through a two-part seminar workshop series, co-facilitated by the three librarians from the institutional team who had completed training from the *All of Us* Academic Libraries Program. The first workshop introduced learning community members to the *All of Us* Research Program and its Researcher Workbench. The second workshop featured live demonstrations on how to build cohorts and datasets within the Workbench, and provided an interactive space for discussing how the *All of Us* platform could be integrated into library services, research consultations, and information literacy instruction.

The faculty and student learning communities were connected by having faculty participants serve as mentors to students in the student learning community. The team sent out a formal call to join the learning communities by sharing an email and flyer with professional contacts, in campus newsletters, and in classes to increase reach to students. The promotional materials indicated the anticipated time commitment for participants and expected deliverables and directed prospective participants to complete a brief application. Faculty learning community participants were informed they would be expected to mentor up to two students in a meeting of at least one hour, once a month, and complete at least one deliverable (conference proposal, manuscript, etc.) by the end of the community. Student participants were informed that they would meet regularly with their mentor to assist with their research project and to develop a poster to present at the culminating learning community symposium, to occur during a joint meeting at the end of the semester.

Faculty and student learning community meetings lasted four hours. As faculty were new to *All of Us*, between the first and second meeting, they were asked to explore the Data Browser to become more familiar with the dataset. By the second meeting, they would develop a research topic, which they would refine into research questions at that meeting. By the third meeting, participants were expected to have designed their study so they could begin developing their Workbench and analyzing data. Students, being novice researchers, were tasked with reviewing content about the research process and engaging in guided exploration of the data, in addition to their meetings with their mentors.

Author ZM led the curriculum and pedagogical tool design for faculty and student meetings, based on some existing *All of Us* resources, his experience learning the dataset, and, for students, his experience teaching research methods. The librarian authors further expanded on and enhanced the curriculum based on resources and guidance received from their *All of Us* Academic Libraries Program. For example, the Data Scavenger Hunt Activity [19], created by the *All of Us* Academic Libraries Program, was adapted and used in all three learning communities to give participants an overview of the data types and help them explore potential questions that could be addressed using the *All of Us* data.

In addition to using resources shared through the two grant programs, the team created several pedagogical tools (see Appendices) such as a Group Resume (see Appendix B), and a Research Process Jig-Saw (see Appendix E) to facilitate collaborative learning within the communities. Table 1 provides a detailed overview of the activities conducted within each learning community.

**Table 1:** Summary of Library, Faculty, and Student Learning Communities: Participation, Activities, and Outcomes

	Library Learning Community	Faculty Learning Community	Student Learning Community
**Active dates**	Summer 2024	Spring and Summer 2024	Spring 2024
**Number of meetings**	2	3	3
**Number of Participants**	19	9	17
**Discipline of Attendees**	Liaison Librarianship Archives Institutional Repositories Digital Scholarship Interlibrary Loan Library Administration Web Services	Applied Data Science Communicative Disorders and Sciences Counselor Education Industrial and Systems Engineering Nursing Public Health	Applied Mathematics Biomedical Engineering Counselor Education Engineering Management Human Factors and Ergonomics Research and Experimental Psychology Social Work
**Topics discussed**	Introduction to the *All of Us* Research Program and the Researcher Workbench Live demonstration building cohorts and datasets Exploration of how the *All of Us* platform could be integrated into library services, research consultations, and information literacy instruction.	Introduction to the *All of Us* Research Program and the Researcher Workbench Exploration of biomedical research Introduction to health research questions. Introduction to Jupyter Notebook, R, and Python Discussion of integrating *All of Us* within existing research agendas	Introduction to the *All of Us* Research Program and the Researcher Workbench Exploration of research and the research process Discussion of social justice in research as a UBW
**Pedagogical Tools**	Scavenger hunt Instruction on Researcher Workbench Group discussion (See Appendix A)	Group resume (see Appendix B) Scavenger Hunt. LMS course Mini-seminar videos created by faculty Workspace Creation Group Rotations (see Appendix C)	Group resume Shared LMS course Reflection on researcher identity (See Appendix D) Research Process Jig-saw (see Appendix E) Scavenger Hunt
**Modality**	First meeting hybrid Second meeting online	Spring 2024 In person and online Summer 2024 Online	In person and online Third meeting in person with FLC
**Metrics of Success**	Attendance and engagement levels at seminar series Number of library professionals who sign up for access to the Researcher Workbench	Number of submitted research products (conference proposal, grant proposal, manuscript, etc.)	Completion and presentation of poster at culminating symposium
**Success**	Including the three facilitating librarians, a total of 10 library professionals at SJSU (approximately 14% of all library employees) successfully gained access to the *All of Us* Researcher Workbench.	By the end of the award period, 100% of faculty had at least 1 submitted research project, with some faculty completing two or three products.	100% of students developed and presented at the culminating student research symposium.

## DISCUSSION

These learning communities were effective in building capacity for research data services within the library while cultivating interdisciplinary research teams across campus and creating new opportunities for student and faculty research. The library learning community was effective in building skills for research data support across several units in the library, directly supporting its objective to build institutional capacity within the SJSU Library for data intensive research by developing the data knowledge and skills of library professionals. Participant roles included liaison librarian, archivist, institutional repository coordinator, digital scholarship librarian, and web developer, setting the foundation for collaboration across functional units within the library to build and expand research data services. This aligns with Condon et al.’s recommendation [20] that cultivating internal champions is essential for launching emerging services within existing staffing constraints. The library learning community provides a means for identifying and fostering these internal champions. The library has built on this program by adopting the learning community model in order to build capacity for instruction in emerging literacies such as AI and data literacy. In addition, the recent hiring of a dedicated Digital & Data Literacy Librarian has continued the momentum for data services at our institution and provides new opportunities to build capacity in this area.

The learning community approach is well-suited for the specific challenges of teaching-intensive institutions, and it is no coincidence that this has become a well-established model for capacity building and faculty development on our campus. On campuses where faculty are under pressure to produce research while shouldering heavy workloads in teaching or librarianship, learning communities can provide opportunities and incentives to set aside precious time for shared, faculty-directed learning and development. While this approach cannot resolve every challenge a researcher may encounter when working with *All of Us* data, it can help mitigate some barriers. In this case, the learning community achieved its goals by strengthening participants’ skills to use the Workbench and by encouraging collaboration between health sciences researchers and data science-oriented colleagues. For example, these collaborations have already produced two publications coauthored by healthcare ergonomics and data science researchers.

More broadly, centering the development of research data services around extended, focused work with small communities of researchers, students, and library employees is an efficient way of building capacity for less-resourced institutions that departs from assessment and program design strategies common at research-intensive institutions [21]. Rather than building towards a model of service maturity that outpaces available resources, the learning community approach foregrounds not only the specific needs, but also the knowledge and experience of faculty and staff in our specific context. Learning communities provide a setting to learn from each other, build empathy, and foster collaboration across different institutional roles. For library participants, learning communities may be a unique opportunity to understand the needs of researchers and work towards research data services that are responsive to institutional contexts and constraints.

The present case is unique in that the institutional team received two awards in quick succession, which propelled the communities forward. Although similar awards exist, the NLM award has been discontinued and the *All of Us* Institutional Champion Award has been reduced due to funding challenges at the NIH. In addition to these now reduced award opportunities, institutional teams looking to replicate the learning community structure described in the present case presentation may also consider internal funding opportunities, as one of the strengths of the learning community approach is comparatively lower costs, given the collaborative, self-contained nature of the learning community model.

In conclusion, health sciences research does not only happen at medical schools, just as health data is not only utilized by medical scientists. Research in many fields may utilize health data, and the learning community approach leverages dispersed expertise on campus to meet existing needs. Further, learning communities are highly compatible with the culture, teaching, and research needs of stakeholders at teaching institutions.

## Data Availability

There are no data associated with this article.

## Supplemental Files

**Appendix A:** Group Discussion – Library Learning Community

**Appendix B:** Group Resume

**Appendix C:** Workspace Creation Group Rotations – Faculty Learning Community

**Appendix D:** Reflection on Research Identity-Classroom Discussion Activity

**Appendix E:** Research Process Jig-saw – Student Learning Community
