# Supplementary material for: Fostering collaboration through learning communities: a case report on engaging with All of Us data among library professionals, faculty, and students
Source: J Med Libr Assoc. 2026 Jul 14;114(3):315–22. doi: 10.5195/jmla.2026.2335 (PMC13367310; doi:10.5195/jmla.2026.2335)
Supplement: Supplementary file 1 — Appendix A: Group Discussion – Library Learning Community [file jmla-114-3-315-s01.pdf]

Appendix A

Group Discussion – Library Learning Community

Discussion Prompts:

How can we use and support *All of Us* within the library and through our individual professional roles?

Consider the following areas as you discuss:

- Dataset promotion
- Educational initiatives
- Partnerships and collaborations
- Sustainable support models
- Ethical use and responsible data practices
- Integration within the library’s data services program
