# Supplementary material for: Fostering collaboration through learning communities: a case report on engaging with All of Us data among library professionals, faculty, and students
Source: J Med Libr Assoc. 2026 Jul 14;114(3):315–22. doi: 10.5195/jmla.2026.2335 (PMC13367310; doi:10.5195/jmla.2026.2335)
Supplement: Supplementary file 2 — Appendix B: Group Resume [file jmla-114-3-315-s02.pdf]

Appendix B

Group Resume

Instructions:

In groups of 3-5, share who you are and your story. Use the list of questions below as inspiration to guide your conversation. Then, based on your discussion, create a group resumé that captures the range of your group's expertise and experience using the template here:

- What educational or professional background, training or lived experiences do you bring that is relevant to the field of health research?
- What are your research interests? What specific research interests do you pursue within the realm of health equity?
- Why do you feel passionately about these topics?
- What methodological expertise/research experience do you bring?
- Beyond your academic or professional pursuits, what hobbies or talents do you have that contribute to your work or personal growth?
- Share a "boring fact" about yourself—a mundane detail that others might find surprising or amusing.
- What are some unique perspectives or experiences you bring to the learning community that others might benefit from knowing?
- What are your aspirations for your involvement in this learning community, and how do you hope to contribute to and learn from your peers?
