# Supplementary material for: Fostering collaboration through learning communities: a case report on engaging with All of Us data among library professionals, faculty, and students
Source: J Med Libr Assoc. 2026 Jul 14;114(3):315–22. doi: 10.5195/jmla.2026.2335 (PMC13367310; doi:10.5195/jmla.2026.2335)
Supplement: Supplementary file 3 — Appendix C: Workspace Creation Group Rotations – Faculty Learning Community [file jmla-114-3-315-s03.pdf]

Appendix C

Workspace Creation Group Rotations – Faculty Learning Community

In small groups, provide the allotted time to address the following prompts. Facilitators move through groups to answer questions and support idea development.

Rotation 1 (20 minutes):

- Share the ideas that you have for your research project with the *All of Us* Data
- Add to the project ideas chart in our group resume!
- Identify any immediately pressing needs/questions

Rotation 2 (10-15 minutes):

- Determine the research questions that you will use to guide your research project

Rotation 3 (40 minutes):

- Create your *All of Us* Workspace
- If working in teams, consider copy/pasting the questions into a Google Doc to work together (one person can complete and then share the workspace)
- Share the workspace with your mentees

Rotation 4 (20 minutes):

- Create the cohort and dataset necessary for your project (Hint: Use the data browser to help support this effort)

Rotation 5 (15 minutes):

- Identify next steps for the project
- Identify what tasks to share with mentees (consider approx. 4 hours per week)
