# Supplementary material for: Fostering collaboration through learning communities: a case report on engaging with All of Us data among library professionals, faculty, and students
Source: J Med Libr Assoc. 2026 Jul 14;114(3):315–22. doi: 10.5195/jmla.2026.2335 (PMC13367310; doi:10.5195/jmla.2026.2335)
Supplement: Supplementary file 4 — Appendix D: Reflection on Research Identity-Classroom Discussion Activity [file jmla-114-3-315-s04.pdf]

Appendix D

Reflection on Research Identity- Classroom Discussion Activity

Students work in small groups to reflect on and discuss their identities as developing researchers before sharing insights with the larger learning community.

Discussion prompts:

- Share the experience you have had with research in the past, and what you hope to do in the future.
- Discuss your emerging areas of interest and specialization.
- Identify the areas you want to focus on and need additional support
- Consider how your mentor can best support your growth in these areas
