# Supplementary material for: Fostering collaboration through learning communities: a case report on engaging with All of Us data among library professionals, faculty, and students
Source: J Med Libr Assoc. 2026 Jul 14;114(3):315–22. doi: 10.5195/jmla.2026.2335 (PMC13367310; doi:10.5195/jmla.2026.2335)
Supplement: Supplementary file 5 — Appendix E: Research Process Jig-saw – Student Learning Community [file jmla-114-3-315-s05.pdf]

Appendix E

Research Process Jig-saw – Student Learning Community

A *Research Process Jig-saw* is an active-learning classroom activity that uses the jigsaw teaching strategy to help students understand the stages of the research process.

Instructions:

Step 1: present the research process to students; break research process into parts

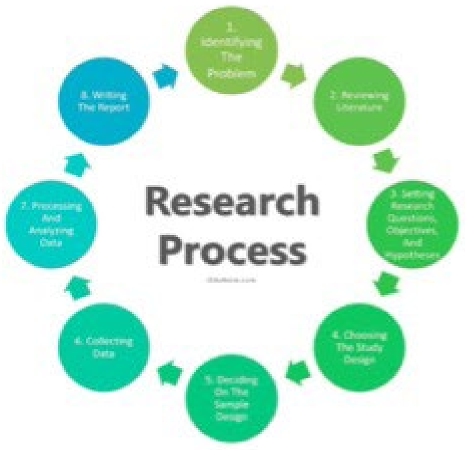

Step 2: Assign each part to a different small group (“expert groups”)

Explore the steps of the research process assigned to your group. Provide a working definition of what the step means, and the type of activities you might do in the step.

Group 1

- Identifying a problem
- Reviewing the literature

- Setting research questions, purpose/objective, hypotheses

Group 2

- Choosing the study design
- Deciding on the sample design
- Collecting data

Group 3

- Analyzing and processing data
- Writing up a report

Step 3: Re-form into mixed groups ("jigsaw groups")

Each new group contains one "expert" from each stage. Members teach one another what they learned.

Step 4: Groups assemble the whole "puzzle"

By sharing their expertise, the group reconstructs the entire research process, just like assembling a jigsaw puzzle.

Why do we use it in the student learning community?

- Encourages collaborative learning
- Helps students recognize research as a multi-step, iterative process
- Ensures each student engages deeply with at least one part of the process
- Supports peer teaching, which improves retention
